# Using medical specialty and selection criteria clusters to study specialty selection by Israeli medical students

**DOI:** 10.1186/s12909-017-0854-y

**Published:** 2017-01-18

**Authors:** Yoram G. Weiss, Rachel Yaffa Zisk-Rony, Howard Tandeter, Uriel Elchalal, Alex Avidan, Josh E. Schroeder, Charles Weissman

**Affiliations:** 1 Department of Anesthesiology and Critical Care Medicine, Hadassah-Hebrew University Medical Center, Hebrew University – Hadassah School of Medicine, Kiryat Hadassah, POB 12000, Jerusalem, 91120 Israel; 2 Department of Family Medicine, Ben Gurion University Joyce and Irving Goldman School of Medicine, Be’er Sheva, Israel; 3 Department of Obstetrics and Gynecology, Hadassah-Hebrew University Medical Center, Hebrew University – Hadassah School of Medicine, Jerusalem, Israel; 4 Hebrew University – Hadassah Henrietta Szold School of Nursing, Jerusalem, Israel; 5 Department of Orthopedic Surgery, Hadassah-Hebrew University Medical Center, Hebrew University – Hadassah School of Medicine, Jerusalem, Israel

**Keywords:** Medical students, Medical education, Residency, Medical specialty selection, Career choice, Medical students, Internship, Choosing a medical specialty

## Abstract

**Background:**

During their final year of medical school, Israeli students must consider which specialty to choose for residency. Based on the vocational counseling literature we presumed that choices are made by selecting from a cluster of related specialties while considering professional and socio-economic issues.

**Methods:**

Questionnaires distributed to final-year medical students at two Israeli medical schools ascertained inclinations toward various medical specialties and the importance of various selection criteria. Analysis focused on seven specialties where >20% of students reported they had positive inclinations. For each such specialty, the specialty and selection criteria query were compared using unpaired two-tailed Student’s t-tests to determine differences between students with positive inclinations toward the specialty with those not so inclined. These data were placed in tables, with the significant differences highlighted to facilitate visual recognition of cluster patterns.

**Results:**

Completed questionnaires were obtained from 317 of 455 students. Students often had positive inclinations toward more than one specialty (specialty clusters) associated with a group of selection criteria (selection criteria clusters). For example, interest in internal medicine was clustered with interest in internal medicine subspecialties, cardiology and research. Furthermore, there was a “reciprocal” aspect to some specialty cluster patterns. For example, those interested in internal medicine had little interest in surgical specialties. Selection criteria clusters revealed occupational interests and socio-environmental factors associated with the specialty clusters. For example, family medicine, which clustered with pediatrics and psychiatry, had a sub-cluster of: Bedside specialty with family orientation affording long-term patient care. Another sub-cluster was time for childrearing and family, only daytime work and outpatient care. Clusters also revealed students’ perceptions that differed from expected: Cardiology is changing from a cognitive to a procedure-oriented subspecialty, clustering not only with internal medicine and its subspecialties but also with emergency medicine, surgical subspecialties and anesthesiology.

**Conclusions:**

The concept that career choice involves selecting from a cluster of related specialties provides information about the specialties students might be considering. Moreover, students are not only looking for individual aspects of a specialty, but for a package including clusters of socio-economic and occupational features. Practically, examining clusters can help in career counseling of medical students and assist residency program directors in marketing their specialties.

**Electronic supplementary material:**

The online version of this article (doi:10.1186/s12909-017-0854-y) contains supplementary material, which is available to authorized users.

## Background

The selection of a medical specialty for residency training and a career by medical students has a major influence on the nature of the future physician workforce. Therefore, to formulate physician workforce policy, healthcare leaders and policymakers, along with residency program directors and department chairs, require objective information about students’ selections and the selection process. During their final year of medical school, Israeli medical students must begin to seriously consider which specialty to choose for residency training and a career. Although the final decision is usually made during the subsequent rotating internship year, the sixth year affords elective time to explore a variety of specialties and subspecialties not included among the required clinical rotations. This specialty selection process involves students formulating a plan for their future by examining both their professional interests and personal situations [1, 2]. They must weigh the relative positive and negative influences of a variety of selection criteria and match them with the characteristics of the various specialties. Although choosing a specialty is often considered a process of *selection*, career choice also has a component of *elimination*, wherein a person progressively eliminates certain alternatives from further consideration [3, 4]. This process of elimination or circumscription involves contextual influences, such as gender, socio-economic valuation (e.g. prestige, income) and work-life balance [5].

It is generally thought that career choices are made by selecting a career from a cluster of related vocational interests [6]. Career clusters are a group of occupations and specialties with similar knowledge requirements, competencies and skill sets. Grouping career possibilities into clusters provides a way of exploring a variety of occupational options. The constituents of these career clusters are determined by a variety of factors including the nature of the occupation (its organization, required skill set and environment) and the requisite personality attributes, plus the individual’s self-determination, skills and psychosocial needs [7–10]. The supposition is that choosing a career will be facilitated by exploring a broad group of occupations allowing the student to examine his/her options and then narrow the decision, based on interest, abilities, social values and personality. The overall assumption is that this process will improve vocational success and satisfaction. The ultimate choice from among the careers in the cluster is accomplished by carefully examining each of the various possibilities. Examining possibilities is often done by gaining practical experience and then determining how potential choices match with expectations and self-identity. In choosing a medical specialty, this process frequently occurs during required and elective clerkships, as well as during career counseling sessions and conversations with residents and more senior physicians [11, 12]. Other factors that also influence the decision include interests, perceived specialty characteristics, lifestyle, financial considerations, the health care environment and the choice process itself [13].

Many studies examined the relationship of medical students’ interest in a single medical specialty and correlating this interest with the relative importance of various selection criteria [14–16]. However, these studies often did not explore the overlap of interests in related specialties and as such did not provide specific information on medical specialty career clusters. Therefore, this study extends these previous observations by exploring the grouping of specialty interests to determine clustering patterns. Furthermore, the study examined the association of these specialty clusters with selection criteria clusters, i.e. it sought to ascertain whether each specialty cluster was related to a specific cluster of selection criteria. The overall goals were to introduce the concept of clustering used in vocational career selection to medical educators, residency program directors and departmental chairs involved with selection by medical students of a career specialty and examine the specialty/selection criteria clusters among Israeli medical students. A dataset containing information about the interests of Israeli final (sixth) year medical students in the various medical specialties and their assessments of the relative importance of the various criteria used in medical career selection was used [17]. This dataset provided a robust picture of medical specialty-selection criteria clusters which in turn should provide medical educators and residency program directors with added information to define target populations at which to direct recruitment strategies for the various specialties.

## Methods

To study medical specialty-selection criteria clusters we used a dataset containing information gathered from a questionnaire distributed to 6^th^ (final) year medical students (Additional file 1 Appendix). The questionnaire [17] was designed to educe from the students various aspects of choosing a medical specialty. Its design was based on the AIUAPR (awareness, interest, understanding, attitudes, purchase and repeat purchase) and other models of consumer behavior [17]. In this marketing research-derived model we considered the specialties as products to be sold to students (consumers) by the medical departments (vendors) [15].

Three sections of the questionnaire elicited information on the students’:

1. Inclinations toward pursuing a career in various medical specialties (19 were listed in the questionnaire). Research, although not a specialty per say, was included to gauge the academic interests of the students. (Additional file 1 Appendix – Section 1)
2. Understanding of how each of 27 criteria influences their selection of a medical specialty (these criteria were chosen from the career choice and medical education literatures). (Additional file 1 Appendix – Section 2)
3. Demographic information – gender, age, marital status (Additional file 1 Appendix – Section 5)

The first two sections, which examined inclinations and selection criteria, used 5-point Likert Scales. The other two sections of the questionnaire were not used in this study since they elicited information about the students’ perceptions and their level of interest in pursuing a career among a group of six selected specialties. This information has been reported previously in a study examining student’s perceptions of six key medical specialties [17, 18].

Prior to it being used in the study, the questionnaire was subjected to two small consecutive preliminary studies of fifteen 6^th^-year medical students each, aimed at testing user friendliness, identifying errors and determining whether changes were required (the pilot data were not included in the study). The major problem recognized during the preliminary studies was that it was necessary to limit the number of medical specialties and selection criteria in some of the sections to allow the students to complete the questions within 15–20 min.

The final version of the questionnaire was distributed to 4 consecutive classes (2007–2010) of 6^th^-year students at the Hebrew University – Hadassah School of Medicine in Jerusalem and the 2010 class of the Ben Gurion University Joyce and Irving Goldman School of Medicine in Be’er Sheva. Portions of these data have been reported in a methodological validation paper, a report of student’s perceptions of six key specialties and a report of the differences between male and female interests in orthopedic surgery [17–19].

This study received approval from the Institutional Review Board of the Hadassah Medical Organization. The completion of the questionnaire by the student was considered as tacit consent.

### Data analysis

Data were entered into Microsoft Excel (Microsoft, Redmont, WA)) spreadsheets and analyzed with Systat Version 12 (Systat Inc. San Jose, CA). When the Likert Scale results were considered continuous variables, statistical analyses were performed using all 5 points. When used as categorical variables the Likert Scale results were compressed into three categories, (the two points representing negative tendencies and the two points representing positive tendencies were combined). The proportion (percentages) of total responses for each of the three categories (positive tendency, middle point and negative tendency) was then computed.

The initial step in the analysis was to better understand the students’ responses to the questionnaires, specifically the relationships between their answers to the various queries. The positive tendency Likert Scale medical specialty (Background) and selection criteria (Methods) data (as ordinal data) each underwent factor analysis (principal components analysis) using varimax rotation with set eigenvalues of ≥ 1.0. This analysis allowed us to “reduce” the number of variables (i.e. specialties and selection criteria) by placing them into categories (factors). This helped us to identify related specialties and related selection criteria. An eigenvalue ≥1.0 indicates that an individual variable (i.e. specialty or selection criteria) belongs to the larger group (i.e. the factor).

The positive tendency Likert Scale medical specialty (Background) and selection criteria (Methods) data were then subjected to hierarchal cluster analysis, an analysis tool that places similar observations into groups called clusters. Cluster analysis complements factor analysis but they differ; cluster analysis categorizes data into groups while factor analysis simplifies or “reduces” data so that, for example, in future studies only one instead of two similar questions need be asked.

Once the factor and cluster analyses of the entire dataset provided a better understanding of how the various queries on the questionnaire were associated, we focused on data from specialties where greater than 20% of the students replied that they had a positive/very positive inclination (Likert data used as categorical data). For each such specialty, each of the specialty and selection criteria queries were compared using unpaired two-tailed Student’s t-tests to determine whether there were differences between the students with positive inclinations toward the specialty with those not so inclined. These data were then put into tables, where the variables were grouped according to the results of the factor analyses, with the significant differences highlighted (bolded) to allow for visual recognition of cluster patterns.

To further confirm specialty cluster patterns, the data (as ordinal data) for specialties where > 20% of the students had a positive/very positive inclination were initially subjected to univariate analysis and those specialties with an r > 0.1 were then included in a backward multivariate regression analysis. The dependent variable was the specialty being studied (e.g. internal medicine and the independent variables were the other specialties (e.g. cardiology, pediatrics). The aim of this analysis was to determine associations between positive/very positive inclinations toward the specialty under study and the other specialties. The regression analysis results were compared with the results obtained with the unpaired Students t-tests.

The demographic data was analyzed using Student’s t-tests to compare continuous variables. Chi-squared analysis was performed for binomial responses. Statistical significance was set at *p* < 0.05.

## Results

Questionnaires were distributed to 455 students and responses obtained from 317 (70%). The good response rate was due to our distributing the questionnaires at the end of a lecture where all the 6th year students were present and giving them time to fill out the questionnaires. Fifty-three percent (*n* = 167) of the 317 students were women. Fifty-two percent of the 317 students were married, 45% were single and the remainder divorced or widowed.

**Fig. 1:**
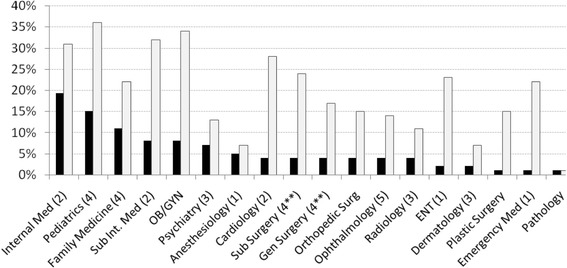
The inclinations of the medical students (*n* = 317) toward the various medical specialties (*gray bars*) are displayed on the same graph as the proportion of certified physicians in the corresponding specialties in Israel (*n* = 13,279, *black bars*). The numbers after some of the specialties are the results of the factor analysis. These show five factor groups. Sub Int Med – Internal medicine subspecialties (e.g endocrinology, gastroenterology, etc.); Sub Surgery – Surgical subspecialties (e.g. urology, cardiothoracic surgery); ENT – otolaryngology. ** - a reciprocal (negative) member of factor group #4

The positive inclinations of the Israeli students towards the various specialties are graphed along with the distribution of specialists practicing in Israel in each of these specialties (Fig. 1). Factor analysis demonstrated 5 factors (see Fig. 1), while cluster analysis revealed 2 clusters. Among the factors was one incorporating internal medicine, internal medicine subspecialties and cardiology. Similarly, cluster analysis revealed a cluster of internal medicine and internal medicine subspecialties. Another factor was pediatrics and family medicine with reciprocal members: general surgery and surgical subspecialties. Likewise, cluster analysis showed general surgery and surgical subspecialties to be a cluster.

The students’ assessments of the importance of the various selection criteria by the students are found in Table 1. The factor analysis identified 6 factors, 3 of them major groups of selection criteria. One factor group of selection criteria included criteria indicating interest in surgical and procedural specialties such as: specialties providing immediate satisfaction, specialties with much action, performing procedures and time in the operating room. This factor corresponded to the results of cluster analysis of specialties with much action, performing procedures and time in the operating room. The second factor group of selection criteria involved personal issues such as time with family, time for childcare, controllable lifestyle and working only during the daytime. This factor corresponded to cluster analysis results of time with family, time for childcare and controllable lifestyle. The third factor group involved professional characteristics of the specialty such as direct patient care, bedside care and long-term care.

**Table 1:** Criteria for selecting specialties

Selection criteria	(*n* = 317)
Interesting Specialty	91%
Immediate Satisfaction	53% (1)*
Performing Procedures	48% (1)
Much Action	37% (1)
Operating Room Time	35% (1)
Controllable Lifestyle	71% (2)
Work only during the Daytime	29% (2)
Work Outside the Hospital	12% (2)
Specialty with Long-term Care	40% (3)
Prestigious Specialty (colleagues)	13% (4)
Prestigious Specialty (population)	11% (4)
Work Only in Hospital	20% (5)
Family Orientation	38%
On-Calls as an Attending	35%
Medical Administration	23%
Without Long-term Care	14%

Percentages are important/very important answers on a 5 point-Likert Scale

*Values in parenthesis are the factor groupings obtained from factor analysis

**Fig. 2:**
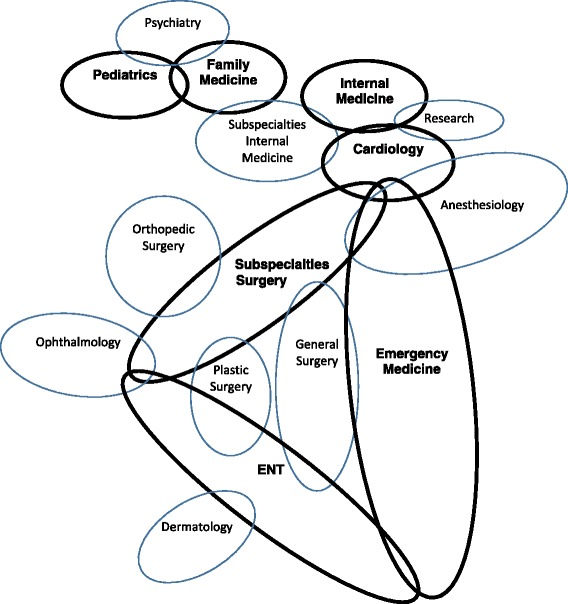
The specialty clusters are graphically displayed including overlapping interests between the clusters

The results of the analyses of seven specialties where >20% of the students replied that they had positive/very positive inclinations are found in Tables 2 and 3 (medical specialties) and Tables 4 and 5 (surgical specialties). The bolded results in the Tables 2 and 3 highlight the specialty and selection criteria cluster patterns. A graphic representation of the results is found in Fig. 2. Students often had positive/very positive inclinations toward more than one specialty (specialty clusters; Tables 2 and 4) that were associated with a group of selection criteria (selection criteria clusters; Tables 3 and 5). An example of an intra-specialty cluster pattern was that students interested in internal medicine were also interested in internal medicine subspecialties, cardiology and research. However, this analysis revealed a “reciprocal” aspect to the cluster pattern. This indicated that these students were surgically averse and had little interest in general surgery, surgical subspecialties, orthopedic surgery, ENT, plastic surgery and ophthalmology. These results were echoed by the multiple regression analysis which showed an inclination towards internal medicine positively associated with cardiology and subspecialties of internal medicine and negatively associated with surgical subspecialties and ophthalmology (Table 2).

**Table 2:** Characteristics of students interested in various medical specialties

			Internal		Family		Emergency	
	Pediatrics	Others	Medicine	Others	Medicine	Others	Medicine	Others
	(*n* = 110; 36%)	(*n* = 207; 64%)	(*n* = 94; 30%)	(*n* = 223; 70%)	(*n* = 68; 21%)	(*n* = 249;79%)	(*n* = 67;21%)	(*n* = 250;79%)
Women/Men	66%/34%*	46/54%	53%/47%	53/47%%	**66%/34%‡**	39%/61%*	46%/54%	55%/45%
Single	47%	49%	**64%‡**	47%	43%	54%	61%	50%
Internal Medicine	32%	32%	100%*	0%	33%	30%	38%	28%
Pediatrics	100%*	0%	33%	37%	56%*	31%	27%	39%
Family Medicine	33%*	14%	23%	21%	100%*	0%	15%	23%
Sub Int. Medicine	34%	32%	68%*	17%	**40%‡**	30%	33%	32%
Cardiology	25%	29%	48%*	19%	20%‡	**30%**	42%*	23%
Psychiatry	**18%‡**	10%	15%	12%	**20%‡**	11%	11%	14%
Anesthesiology	5%	9%	9%	7%	3%	8%	18%*	4%
General Surgery	7%*	**22%**	12%*	**18%**	5%*	**20%**	31%*	12%
Sub Surgery	11%*	**30%**	12%*	**29%**	6%‡	**29%**	**32%‡**	22%
Plastic Surgery	10%*	**17%**	8%*	**18%**	9%*	**17%**	**25%‡**	12%
Orthopedic Surg	6%*	**20%**	3%‡	**20%**	2%*	**19%**	19%	14%
Ophthalmology	12%	14%	7%*	**17%**	11%	14%	20%	12%
ENT	17%	26%	11%*	**28%**	16%‡	**25%**	30%*	21%
Dermatology	7%	7%	8%	7%	3%	8%	8%	7%
OB/GYN	31%	34%	23%	38%	38%	32%	30%	34%
Radiology	11%	10%	6%	13%	13%	10%	11%	11%
Emergency Med	16%	25%	28%	19%	15%	24%	100%*	0%
Research	14%	10%	14%*	10%	13%	11%	13%	11%
	*p < 0.001 vs Others					‡*p* < 0.05 vs Others		
Multiple	Specialties: *r* = 0.45		Specialties: *r* = 0.75			Specialties: *r* = 0.48	Specialties: *r* = 0.52	
Regression	+Family Medicine: *p* < 0.002		+Cardiology: *p* < 0.0001			+Dermatology: *p* < 0.031	+General Surgery: *p* < 0.001	
	-General Surgery: *p* < 0.004		+Sub Int Med: *p* < 0.0001			+Sub IntMed: *p* < 0.007	+Anesthesiology: *p* < 0.0001	
	-Pathology: *p* < 0.011		-Sub Surgery: *p* < 0.025			+Psychiatry: *p* < 0.002	+Cardiology: *p* < 0.001	
			-Ophthalmology: *p* < 0.01			+Gynecology: *p* < 0.009	+ENT: *p* < 0.0001	
						+Pediatrics: *p* < 0.001	+Sub Surgery: *p* < 0.011	
	**+** Positive Correlation		- Negative Correlation					

Sub Int. Medicine – Subspecialties of Internal Medicine (e.g. endocrinology, gastroenterology, pulmonology, etc.)

Sub Surgery – Surgical Subspecialties (e.g. urology, cardiothoracic surgery, vascular surgery, etc.)

ENT - otolaryngology

Specialties are arranged according to the results of the factor analysis (Fig. 1)

Values are the percentage of strong/very strong inclinations on the 5-point Likert scale

Bold values are significantly greater than the other member of the pair (unpaired *t*-test)

**Table 3:** Characteristics of the selection criteria of students interested in various medical specialties

			Internal		Family		Emergency	
	Pediatrics	Others	Medicine	Others	Medicine	Others	Medicine	Others
	(*n* = 110; 36%)	(*n* = *n* = 207; 64%)	(*n* = 94; 30%)	(*n* = 223; 70%)	(*n* = 68; 21%)	(*n* = 249;79%)	(*n* = 67;21%)	(*n* = 250;79%)
Interesting Specialty	92%	91%	89%	66%	89%	92%	**96%**	90%*
Direct Aid to Patients	79%	77%	78%	78%	79%	78%	78%	78%
Beside Specialty	**82%**	69%*	**89%**	66%‡	**83%**	71%‡	78%	73%
Long-term Care	**48%**	36%‡	**50%**	36%*	**70%**	41%‡	30%	43%*
Direct Patient Care	**72%**	57%‡	60%	68%*	82%	57%	51%	65%
Family Time	**90%**	75%‡	81%	81%	**88%**	79%*	73%	83%
Controllable Lifestyle	71%	72%	69%	72%	**82%**	69%*	67%	73%
Time to Raise Children	**82%**	65%‡	70%	73%	**88%**	68%‡	66%	74%
Daytime Work Only	**42%**	21%‡	23%	31%	**45%**	24%*	20%	32%
Work Outside Hospital	12%	12%	11%	13%	**25%**	9%‡	11%	14%*
Immediate Satisfaction	52%	57%	38%	60%*	40%	57%*	**73%**	48%‡
Performing Procedures	33%	55%	27%	57%*	23%	**54%‡**	**60%**	44%‡
Much Action	25%	43%*	25%	42%*	22%	**41%‡**	**66%**	28%‡
Operating Room Time	10%	**45%‡**	17%	43%*	14%	**41%‡**	37%	35%
Work Only in Hospital	15%	22%	27%	16%	19%	20%	25%	18%
Academic Opportunities	43%	**57%‡**	**59%**	49%*	27%	59%*	61%	49%
Prestigious Specialty§	8%	15%*	13%	12%	0%	**16%‡**	**21%**	10%*
Prestigious Specialty**	7%	12%	11%	11%	3%	**13%‡**	16%	9%
High Salary	44%	54%	38%	56%*	39%	54%	58%	49%
Clerkship Experience	**50%**	41%*	51%	41%	42%	32%	51%	42%
Family Orientation	**50%**	30%‡	42%	36%	**74%**	22%‡	28%	40%
On-Calls as an Attending	32%	36%	37%	33%	22%	38%*	**57%**	28%‡
Medical Administration	20%	23%	24%	21%	24%	22%	31%	20%
Private Practice	48%	58%	48%	57%	38%	59%*	58%	54%
Without Long-term Care	10%	15%	5%	17%*	11%	14%*	13%	13%

Values are the percentage of important/very important on the 5-point Likert scale

Bold values are significantly greater than the other member of the pair (unpaired *t*-test)

‡*p* < 0.001 vs Other group

§in the view of colleagues

*p < 0.05 vs Other group

**in the view of the population

**Table 4:** Characteristics of students interested in surgical specialties

	Surgical					
	Subspecialties	Others	ENT	Others	Cardiology	Others
	(*n* = 72; 23%)	(*n* = 245; 77%)	(*n* = 71;22%)	(*n* = 246; 78%)	(*n* = 84; 27%)	(*n* = 233; 73%)
Women/men	26%/74%	61%*/39%	38%/62%	58%*/42%	39%/61%	**58%/42%**
Single	50%	52%	51%	45%	55%	51%
Internal Medicine	15%	**35%‡**	14%	35%*	**52%**	22%‡
Pediatrics	17%	**42%‡**	27%	39%	32%	37%
Family Medicine	6%	**26%‡**	13%	23%*	16%	24%
Sub Int. Medicine	19%	**36%‡**	23%	35%	**46%**	27%‡
Cardiology	33%	26%	30%	27%	**100%**	0%‡
Psychiatry	3%	16%*	9%	14%	8%	17%
Anesthesiology	**11%**	6%*	9%	7%	**12%**	6%‡
Sub Surgery	**100%**	0%‡	**45%**	17%‡	**29%**	22%*
General Surgery	**44%**	5%‡	**24%**	14%*	17%	16%
Orthopedic Surg	**42%**	6%‡	**33%**	10%‡	12%	16%
Ophthalmology	**24%**	10%*	**30%**	7%‡	13%	14%
Radiology	13%	10%	16%	10%	8%	11%
ENT	**44%**	17%‡	**100%**	0%‡	25%	22%
Dermatology	8%	7%	**10%**	6%*	8%	6%
Plastic Surgery	**29%**	10%‡	**37%**	8%‡	12%	16%
Emergency Med	29%	20%	**29%**	18%*	**34%**	18%‡
Pathology	3%	1%	3%	0%	1%	1%
OB/GYN	40%	31%	37%	32%	32%	34%
Research	13%	10%	16%	10%	**17%**	9%*
	*p < 0.001 vs Others		‡*p* < 0.05 vs Others			
	Multiple Regression		Multiple Regression		Multiple Regression	
	Specialties: *r* = 0.80		Specialties: *r* = 0.61		Specialties: *r* = 0.65	
	+General Surgery: *p* < 0.0001		+Plastic Surgery: *p* < 0.0001		+Sub Internal Med: *p* < 0.001	
	+Orthopedic Surgery: *p* < 0.0001		+Orthopedic Surgery: *p* < 0.0001		+ENT: *p* < 0.04	
	+Opthalmology: *p* < 0.041		+Ophthalmology: *p* < 0.001		+Sub Surgery: *p* < 0.001	
	+Cardiology: *p* < 0.001		+Emergency Medicine: *p* < 0.0001		+Ophthalmology: *p* < 0.011	
	-Pediatrics: *p* < 0.015		+Cardiology: *p* < 0.048		+Emergency Medicine: *p* < 0.009	
	-Cardiology: *p* < 0.001		+General Surgery: *p* < 0.041		+internal Medicine: *p* < 0.0001	
			-Psychiatry: *p* < 0.039		-Dermatology: *p* < 0.024	
			-Internal Medicine: *p* < 0.018		-Plastic Surgery: *p* < 0.047	

Values are the percentage of important/very important on the 5-point Likert scale

Bold values are significantly greater than the other member of the pair (unpaired *t*-test)

**Table 5:** Characteristics of the selection criteria of students interested in surgical specialties

	Surgical					
	Subspecialties	Others	ENT	Others	Cardiology	Others
	(*n* = 72;23%)	(*n* = 245; 77%)	(*n* = 71;22%)	(*n* = 246; 78%)	(*n* = 84;27%)	(*n* = 233; 73%)
Interesting Specialty	**96%**	90%*	93%	90%	94%	90%
Direct Aid to Patients	83%	76%	76%	78%	75%	80%
Beside Specialty	63%	77%*	64%	76%*	80%	71%
Long-term Care	17%	**48%‡**	28%	43%*	35%	42%
Direct Patient Care	42%	**68%‡**	59%	63%	62%	62%
Family Time	62%	**87%‡**	76%	82%	73%	83%
Controllable Lifestyle	65%	74%	76%	70%	62%	75%*
Time to Raise Children	54%	**79%‡**	70%	73%	62%	76%*
Daytime Work Only	19%	**32%‡**	21%	31%	21%	32%*
Work Outside the Hospital	11%	13%	10%	13%	11%	10%
Immediate Satisfaction	**82%**	44%‡	**75%**	47%‡	56%	53%
Performing Procedures	**90%**	35%‡	**79%**	38%‡	**53%**	46%*
Much Action	**58%**	30%‡	**51%**	33%‡	**43%**	33%‡
Operating Room Time	**79%**	22%‡	**69%**	29%‡	31%	37%
Work Only in Hospital	23%	18%	10%	13%	**24%**	18%*
Academic Opportunities	**65%**	48%*	59%	49%	**66%**	46%*
Prestigious Specialty§	**24%**	9%*	**25%**	9%*	**21%**	10%‡
Prestigious Specialty**	**17%**	9%*	**21%**	7%*	**15%**	10%‡
High Salary	**70%**	44%‡	**69%**	45%‡	49%	51%
Experience during Clerkship	47%	43%	51%	42%	44%	44%
Family Orientation	20%	**43%‡**	32%	39%	29%	41%
On-Calls as an Attending	**42%**	32%*	41%	33%	**41%**	32%*
Medical Administration	27%	21%	**31%**	20%*	29%	20%
Private Practice	**68%**	50%‡	**80%**	47%‡	54%	54%
Without Long-term Care	**21%**	11%‡	21%	11%	9%	15%

Values are the percentage of important/very important on the 5-point Likert scale

Bold values are significantly greater than the other member of the pair (unpaired *t*-test)

*p < 0.001 vs Other group

**in the view of the population

‡*p* < 0.05 vs Other group

§in the view of colleagues

## Discussion

Choosing an educational and eventually an occupational preference results from evaluative self-observation based on a potential career’s requisite intellectual requirements and tasks. This self-observation is often enhanced and constrained, by environment events, social learning, gender expectations and economic considerations [20, 21]. These elements, which were gleaned from various career selection theories, are important to consider when examining medical specialty-selection criteria clusters. Family medicine provides a good example for examining career interest in this context. It was clustered with pediatrics and psychiatry (Table 2) both disciplines whose knowledge and skill sets are part and parcel of the routine practice of family medicine. When selection criteria were examined both positive and negative sub-clusters were identified. Two positive selection criteria sub-clusters were identified. One sub-cluster concerned the nature of the occupation, i.e. a bedside specialty (i.e. direct patient care) with a family orientation, affording long-term patient care. The other sub-cluster, involved socio-environmental factors, i.e. a specialty that allows time with the family and for childrearing thus allowing for work only during the daytime hours and not in the hospital. In addition to these clusters, there was a dominant negative specialty sub-cluster which included procedure/surgery oriented specialties, such as cardiology and surgical specialties, along with associated selection criteria, such as a specialty with much action. The latter is an example of the elimination aspect of career selection. Negative factors have important influences both initially when medical students choose a specialty and also if they decide to switch their selection [22]. These finding are not unexpected nor novel to the Israeli and other national settings, but they add cross-specialty information that complement previous comparisons between students interested and not interested in family medicine [23]. They also give credence to combined family medicine – psychiatry residency programs such as exist in a number of United States hospitals.

The results of this study also demonstrate that in addition to exploring within specialty clustering, it is useful to compare cluster patterns between specialties. This was demonstrated by the interaction between pediatrics and family medicine. Students interested in family medicine were significantly more interested in pediatrics than were the remainder of the students and vice versa. Furthermore, both groups were significantly more interested in psychiatry and less interested in the surgical specialties than those not interested in these specialties. Moreover, there was a moderate overlap among those interested in these two specialties. Of the 110 students interested in pediatrics and 68 in family medicine, 36 were interested in both specialties. Additionally, the students’ interested in pediatrics and family medicine had similar selection criteria sub-clusters; both reported interest in bedside specialties involving direct patient care and providing time to raise children. However, there were also differences between those interested in family medicine and pediatrics. Compared to students interested in pediatrics, those interested in family medicine rated a controllable lifestyle and working outside the hospital as important positive selection criteria, while high salaries were notably less important. This is similar to the results of Japanese medical students who rated work-life balance and rural practice as an important reasons for specializing in family medicine [24]. These results slso demonstrate that although there are many similarities and some overlap among the students inclined to pursue these two specialties, they are functionally separate populations. Other non-Israeli investigators have made similar observations, noting that students interested in pediatrics were more interested in working with children and less interested in caring for adults than those interested in family medicine [25]. Therefore, at least in Israel, different strategies should be employed to recruit students to each of these two specialties.

The cluster approach to examining medical student interests in the various medical specialties provided a number of insights that might affect the healthcare and medical education systems. This was illustrated by students interested in internal medicine and cardiology who had significantly greater interests in academic opportunities and research than students not interested in these disciplines. This contrasted with the students interested in pediatrics and family medicine who were significantly less interested than the other students in pursuing academic opportunities. These results might have implications for the future of medical education and research, especially the importance of having mentors from all medical specialties active and well represented in all medical schools [26–28]. This is especially important for family medicine which in many countries has difficulties recruiting sufficient residents.

The cluster method used in this study revealed results for cardiology and emergency medicine that deviated from the expected. The specialty-selection criteria cluster associated with cardiology, which in Israel is a free-standing specialty and not a sub-specialty of internal medicine, showed that it is changing from a cognitive subspecialty of internal medicine to a procedure-oriented subspecialty [29, 30]. Students interested in cardiology were not only significantly more interested in internal medicine and its subspecialties than were the remainder of the students, but were also more interested in emergency medicine, the surgical subspecialties, and anesthesiology. This surgical inclination likely reflects the complex procedures performed by cardiologists, such as transcutaneous aortic valve implantation, mitral valve clipping and the emergency treatment of acute coronary events. Furthermore, the selection criteria sub-clusters echoed the profile of students interested in pursuing surgical subspecialties and otolaryngology (Table 5, Fig. 2). These findings are important given the increasing demand for interventional cardiologists, who in many countries sub-specialize in cardiology after completing internal medicine residencies [31]. Thought should be given to expanding this pool of potential interventional cardiologists to recruiting among students and residents with a surgical orientation.

Emergency medicine, another specialty that straddles the medical-surgical border, elicited much interest among the students even though it is a nascent specialty in Israel. Notably, these students’ specialty-selection criteria cluster was similar to that of students interested in the surgical subspecialties, i.e. interests in a specialty with immediate satisfaction and performing procedures. Others have made similar observations and concluded that the trauma care component of the specialty is a major attraction for students [32]. However, these responses were somewhat unexpected, since emergency medicine specialists spend much time diagnosing and treating internal medicine and pediatric problems. This incongruity might indicate a lack of familiarity with the nature of emergency medicine practice.

The specialty composition of a country’s physician workforce is determined by a number of factors including the number of residency positions in each specialty and the specialty choices of medical students. The present study examined the latter factor so it was possible to compare the patterns of the students’ specialty inclinations with the composition of the present Israeli specialty workforce. The Israel healthcare leadership does not formally project the future needs for various specialists. Therefore, the supposition made was that the present composition of the physician workforce roughly reflects the general demand for specialists in the near future. Using data published by the Israel Ministry of Health, the comparison showed a moderate overall association (Fig. 1). Generally, the interests of the students paralleled those of the specialty workforce. However, the moderate correlation was attributable to a disproportionate lack of inclination towards psychiatry and anesthesiology and inordinate interests in otolaryngology, plastic surgery and emergency medicine (Fig. 1). These data portend future problems for the healthcare system, especially since psychiatry and anesthesiology are the 5th and 6th largest single specialties, respectively, (when the aggregate of internal medicine subspecialists is not included) and are already suffering from insufficient workforce [33]. The present study demonstrated that students interested in emergency medicine, cardiology and surgical subspecialties also had interests, although weak, in anesthesiology. Thought should be given to cultivating these interests in order to recruit some of these students to anesthesiology.

### Strengths and limitations

Among the strengths of this study is its trans-disciplinary approach. It melds the vocational career concept that career selection involves choosing from a cluster of related vocational interests with methods traditionally used to examine the medical specialty selection process. This conglomeration of ideas resulted in our examining specialty-selection criteria clusters. This approach permitted us to simultaneously examine personal, social and contextual issues, providing insights and associations within and among specialties that add to our understanding of the students’ selection process. Another strength is the study’s breadth, in that it did not focus on the selection process for a single specialty, but instead provides a comprehensive picture that allowed examining the selection criteria pattern for a number of specialties. A limitation of the cluster methodology is that in order to examine the specialty-selection criteria clusters associated with specialties that interest only a few students, large populations of students must be studied to provide sufficient numbers to perform a valid analysis. A limitation of the present study is that it involved students in their final year of medical school. Since some students do not make their final choice until their internship year or possibly later, the results of this study may possibly not reflect their final decisions since career identity change during the course of a person’s career experience [34–38]. Therefore, future studies would be enhanced by correlating such findings with final residency selections and residency completion rates.

## Conclusions

Medical educators, residency program directors, medical department heads and healthcare system leaders worldwide must be cognizant of the professional, cultural and social trendsoperative among students during the specialty selection process. Studies exploring specialty-selection criteria clusters, such as the present one, are thus vital for providing objective data on these issues. Since the composition of specialty-selection criteria clusters likely vary from country-to-country it is important to locally perform studies similar to the present one. In a practical sense, results of such studies should help medical educators and others in the career counseling of medical students, as well as assist residency program directors and medical department heads in marketing their specialties [17]. The latter often operate in a competitive environment where “consumers” (medical students and interns) are looking for a “product” (medical specialty). These “vendors” (the medical departments), thus, need to understand who is their target population and their criteria for purchasing a “product” [17]. Furthermore, the concept that choosing a career involves selecting from among a cluster of related specialties provides information about potential “competitors” i.e. related specialties that the students and interns might also be considering. Moreover, this study adds to previous studies by showing that students are not looking only for individual aspects of a specialty, but for a package (or “solution”) that includes a cluster of socio-environmental, economic and occupational features [38].

## Additional file

Additional file 1: Appendix A – Questionnaire. (DOC 94 kb)
