# Supplementary material for: Using medical specialty and selection criteria clusters to study specialty selection by Israeli medical students
Source: BMC Med Educ. 2017 Jan 18;17:17. doi: 10.1186/s12909-017-0854-y (PMC5241925; doi:10.1186/s12909-017-0854-y)
Supplement: Additional file 1: — Appendix A – Questionnaire. (DOC 94 kb) [file 12909_2017_854_MOESM1_ESM.doc]

APPENDIX A – QUESTIONNAIRE

**Section 1.** Please Complete

Please indicate your degree of interest in each of these specialties

| Specialty | Not at all Interested | Not Interested | Neutral | Interested | Very Interested |
| --- | --- | --- | --- | --- | --- |
|  | 1 | 2 | 3 | 4 | 5 |
| Internal Medicine |  |  |  |  |  |
| Cardiology |  |  |  |  |  |
| Sub Int. Medicine |  |  |  |  |  |
| Pediatrics |  |  |  |  |  |
| Family Medicine |  |  |  |  |  |
| Sub-Specialties of Surgery ( e.g. Urology) |  |  |  |  |  |
| Pathology |  |  |  |  |  |
| Dermatology |  |  |  |  |  |
| General Surgery |  |  |  |  |  |
| Ophthalmology |  |  |  |  |  |
| Orthopedic Surgery |  |  |  |  |  |
| Emergency Medicine |  |  |  |  |  |
| Radiology |  |  |  |  |  |
| Psychiatry |  |  |  |  |  |
| OB/GYN |  |  |  |  |  |
| Anesthesiology |  |  |  |  |  |
| ENT |  |  |  |  |  |
| Plastic Surgery |  |  |  |  |  |
| Research |  |  |  |  |  |

**Section 2.** Please complete

Please indicate how each of 25 criteria influences your choice of a medical specialty.

| Criteria | Not at All Important | Not Important | Neutral | Important | Very Important |
| --- | --- | --- | --- | --- | --- |
|  | 1 | 2 | 3 | 4 | 5 |
| Interesting and Challenging Specialty |  |  |  |  |  |
| Work Only in Hospital |  |  |  |  |  |
| Prestigious Specialty in the View of Colleagues |  |  |  |  |  |
| On-Calls as an Attending |  |  |  |  |  |
| Bedside Specialty |  |  |  |  |  |
| Direct Patient Care |  |  |  |  |  |
| Medical Administration |  |  |  |  |  |
| Academic Opportunities |  |  |  |  |  |
| Much "Action" |  |  |  |  |  |
| Long-term Care |  |  |  |  |  |
| Family Orientation |  |  |  |  |  |
| Work only during the Daytime |  |  |  |  |  |
| Time to Raise Children |  |  |  |  |  |
| Direct Aid to Patients |  |  |  |  |  |
| Controllable Lifestyle |  |  |  |  |  |
| Immediate Satisfaction |  |  |  |  |  |
| Interesting Specialty |  |  |  |  |  |
| Without Long-term Care |  |  |  |  |  |
| Work Outside the Hospital |  |  |  |  |  |
| Prestigious Specialty in the View of Population |  |  |  |  |  |
| Private Practice |  |  |  |  |  |
| Time with Family |  |  |  |  |  |
| Performing Procedures |  |  |  |  |  |
| High Salary |  |  |  |  |  |
| Operating Room Time |  |  |  |  |  |
| Experience during Clerkship |  |  |  |  |  |

**Section 3.** Please rate each specialty as follows: 1 – definitely do not agree, 2- do not agree 3 – neutral 4- agree 5- very much agree

|  | Pediatrics | Orthopedic Surgery | Anesthesiology | General Surgery | OB/GYN | Family  Medicine |
| --- | --- | --- | --- | --- | --- | --- |
| Advanced Specialty |  |  |  |  |  |  |
| Affords Controllable Lifestyle |  |  |  |  |  |  |
| Prestigious Specialty (colleagues) |  |  |  |  |  |  |
| Reasonable ratio of lifestyle vs income |  |  |  |  |  |  |
| Affords Academic Opportunities |  |  |  |  |  |  |
| Interesting and Challenging Specialty |  |  |  |  |  |  |
| Long Working Hours |  |  |  |  |  |  |
| Possible to Work Limited Hours |  |  |  |  |  |  |
| High Salary |  |  |  |  |  |  |
| Opportunity for Private Practice |  |  |  |  |  |  |
| Stressful Specialty |  |  |  |  |  |  |
| Allows for Family Time |  |  |  |  |  |  |
| Boring Specialty |  |  |  |  |  |  |
| Specialty in Crisis |  |  |  |  |  |  |
| Prestigious Specialty (population) |  |  |  |  |  |  |
| Specialty I am Positively Considering |  |  |  |  |  |  |
| **(Section 4)**  Specialty Students are Positively Considering |  |  |  |  |  |  |

**Section 5** Demographics

1. Which Medical School do you attend?_________________________
2. Gender: Female______ Male________
3. Age__________ years
4. Single_______Married_____Divorced_______Widowed_______
